# The microbiological signature of human cutaneous leishmaniasis lesions exhibits restricted bacterial diversity compared to healthy skin

**DOI:** 10.1590/0074-02760150436

**Published:** 2016-04

**Authors:** Vanessa R Salgado, Artur TL de Queiroz, Sabri S Sanabani, Camila I de Oliveira, Edgar M Carvalho, Jackson ML Costa, Manoel Barral-Netto, Aldina Barral

**Affiliations:** 1 Fundação Oswaldo Cruz, Centro de Pesquisas Gonçalo Moniz, Salvador, BA, Brasil; 2 Instituto de Investigação em Imunologia, São Paulo, SP, Brasil; 3 Faculdade de Medicina da Universidade de São Paulo, Hospital das Clínicas, Departamento de Patologia, São Paulo, SP, Brasil; 4 Universidade Federal da Bahia, Hospital Universitário Prof Edgard Santos, Serviço de Imunologia, Salvador, BA, Brasil; 5 Instituto Nacional de Ciência e Tecnologia em Doenças Tropicais, Salvador, BA, Brasil

**Keywords:** microbiome, LCL, wound, healthy skin, high-throughput sequencing

## Abstract

Localised cutaneous leishmaniasis (LCL) is the most common form of cutaneous leishmaniasis characterised by single or multiple painless chronic ulcers, which commonly presents with secondary bacterial infection. Previous culture-based studies have found staphylococci, streptococci, and opportunistic pathogenic bacteria in LCL lesions, but there have been no comparisons to normal skin. In addition, this approach has strong bias for determining bacterial composition. The present study tested the hypothesis that bacterial communities in LCL lesions differ from those found on healthy skin (HS). Using a high throughput amplicon sequencing approach, which allows for better populational evaluation due to greater depth coverage and the Quantitative Insights Into Microbial Ecology pipeline, we compared the microbiological signature of LCL lesions with that of contralateral HS from the same individuals.*Streptococcus*, *Staphylococcus*,*Fusobacterium* and other strict or facultative anaerobic bacteria composed the LCL microbiome. Aerobic and facultative anaerobic bacteria found in HS, including environmental bacteria, were significantly decreased in LCL lesions (p < 0.01). This paper presents the first comprehensive microbiome identification from LCL lesions with next generation sequence methodology and shows a marked reduction of bacterial diversity in the lesions.

Cutaneous leishmaniasis (CL) is broadly divided into three major clinical phenotypes: diffuse CL (DCL), presenting multiple nonulcerative nodules, mucosal leishmaniasis (LM), characterised by destructive mucosal inflammation, and localised CL (LCL), the most frequent manifestation of CL, characterised by single or multiple painless chronic ulcerative skin lesions, developing at the site of the infected sandfly bite (Reithinger et al. 2007, Costa et al. 2009). CL in the New World is caused mainly by*Leishmania (Viannia) braziliensis*, *Leishmania (Viannia) guyanensis*, *Leishmania (Leishmania) amazonensis*, and*Leishmania (Leishmania) mexicana* (Grimaldi Jr et al. 1989). In the Northeast Region of Brazil, LCL and LM are predominantly caused by *L. (V.) braziliensis* (Rosa et al. 1988) and DCL is most commonly caused by *L. amazonensis* (Bittencourt et al. 1989)*.*

LCL ulcers usually present with a slow recovery and may last several months or years in the absence of antiparasitic treatment (Salman 1999). Therapy is broadly accomplished with pentavalent antimonials (Glucantime^®^; Sanofi-Aventis, Brazil) recommended as first line drugs. Therapeutic response to these drugs is usually favourable, presenting variable cure rates (from 60-100%) (Azeredo-Coutinho et al. 2007). The ulcer chronicity, constant environmental exposure, and poor hygiene at the lesion site associated with ground proximity in lesions located in lower limbs, one of the most affected site, may promote polymicrobial infections. Secondary bacterial infections are frequently observed in LCL patients. This condition, in addition to being uncomfortable for the patient, may sometimes impair the healing process of ulcer and require antibiotic treatment (Vera et al. 2002,^2006^, Fontes et al. 2005, Gonçalves et al. 2009).

Prejudicial effects on the healing process have been reported in the presence of a complex bacterial colonisation also in diabetic lesions and of other chronic wounds (Daltrey et al. 1981, Halbert et al. 1992, Davies et al. 2004, Grice et al. 2010, Percival et al. 2010, Scales & Huffnagle 2013). In LCL lesions, it was observed that individuals presenting secondary infection with purulent secretion and harbouring concomitant infections with *Streptococcus pyogenes*,*Staphylococcus aureus*, *Pseudomonas aeruginosa*,*Morganella morganii*, and *Enterococcus durans*presented with a delayed healing process (Isaac-Márquez & Lezama-Dávila 2003). In other study, patients presenting secondary bacterial infection in CL lesions presented lower cure rates to systemic treatment with glucantime compared with patients who did not presenting contamination. Authors suggest decreased effect of glucantime in CL lesions complicated with secondary bacterial infection. *S. aureus* was the most prevalent organism in these contaminated lesions (Sadeghian et al. 2011). The delayed time in CL lesion healing requires repeated treatment cycles, with harmful effects associated with drug toxicity (Mitropoulos et al. 2010).

Naik et al. (2012) evaluated the role of skin microbiota on local immunity using germ-free (GF) and specific pathogen free (SPF) mice in a model of dermal infection induced by *L. major*. GF mice infected intradermally with *L. major* showed smaller lesions with reduced oedema and necrosis compared to SPF mice, however with higher parasite load in the lesion. These outcomes were associated with a smaller production of interferon-gamma and tumour necrosis factor-alpha by cutaneous T-cell and demonstrated the role of skin microbiota in *Leishmania* infection control. Previous studies comparing GF and conventional (microbiota-bearing) mice infected subcutaneously with *L. major* (revised by Lopes et al. 2016) demonstrated GF mice failed to heal lesions and presented higher parasite load at the infection site than conventional mice. The cytokine production profile did not differ in both; however macrophages from GF mice were not as efficient at killing parasites as conventional mice, suggesting an important role of microbiota in macrophage activation (Oliveira et al. 2005). Vieira et al. (1998) also found similar results with GF mice failing to resolve lesions after 13 weeks of *L. major* infection, in contrast with the conventional mice. Despite this, the cytokine profiles were indistinguishable in both groups. GF mice macrophages, albeit capable of producing nitric oxide (NO) in response to leishmanial infection, were no able to destroy the parasites. Absence of the normal indigenous microbiota was suggested as cause of increased susceptibility to *L. major* infection (Vieira et al. 1998).

However, the leishmaniasis microbiome-composition in humans remains unclear. Few studies for bacterial community identification in LCL lesions have been performed. Previous culture-based studies reported *Staphylococcus* spp,*Streptococcus* spp, *Enterococcus* spp,*Pseudomonas* spp, and other opportunistic bacteria in LCL lesions (Vera et al. 2006, Shirazi et al. 2007, Ziaei et al. 2008, Gonçalves et al. 2009). Nevertheless, this culture-based methodology has a strong bias for estimation of bacterial composition since bacteria presenting low relative abundance, fastidious and/or “unculturable” organisms are underestimated (Rhoads et al. 2012, Scales & Huffnagle 2013).

Advanced molecular methods have indicated that only about 1% or 2% of all the skin-colonising bacteria could be cultivated under usual conditions (Gontcharova et al. 2010, Bertesteanu et al. 2014). Furthermore, culture-independent tests revealed a much greater diversity in skin bacterial communities compared to culture-based estimations (Grice & Segre 2011, Rhoads et al. 2012, Scales & Huffnagle 2013). The use of massive molecular methods allows deep insights into the microbiome-composition in general and also in the LCL microbiome because it is more sensitive than culture, as described for other chronic wounds (Rhoads et al. 2012). Our hypothesis is that bacterial communities in LCL lesions differ from those on contralateral healthy skin (HS). Thus, our aim was to characterise the LCL lesion microbiome and to compare it to that found on HS, in the same individuals, using next generation sequencing.

## SUBJECTS, MATERIALS AND METHODS

*Patient characterisation* - The median age from subjects was 35 years (range 20-48 years). Subjects were two women and eight men without significant differences related to gender. Individuals chosen for the study lived in the endemic area an average of 32.3 years (range 8-44 years), most had agricultural activity, and presented ulcerated skin lesions clinically consistent with CL (Costa et al. 2009). Patients were confirmed with CL after positive results in, at least, one of the following tests realised to the inclusion of them in the study: delayed-type hypersensitivity (DTH) with*Leishmania* antigen, parasite isolation in culture, parasite identification in histopathological studies, or a positive *L. braziliensis* polymerase chain reaction (PCR) performed, as previously described (Weirather et al. 2011). No patient had received previous treatment for leishmaniasis or antibiotics in the last month before sample collection.

*Wound samples and HS collection* - Samples were collected in The Health Post of Corte de Pedra, state of Bahia, Brazil, a CL endemic area. Twenty paired swab samples (10 samples collected from LCL lesions and 10 samples collected from contralateral HS) were obtained using sterile technique after wound cleaning with saline and debridement, when a crust was present. The material collected in swab samples was transferred to MO BIO PowerBead Tubes (PowerSoil DNA Isolation kit; MO BIO Laboratories, Inc, USA) and immediately frozen at -80ºC until DNA isolation.

*Management of patients* - After sample collection, patients were treated with intravenous pentavalent antimony Sb^V^ (20 mg/kg/day for 20 days). The lesion outcome was observed up to the healing. Patients who had failure of treatment after the first course and the lesion persisted after 90 days after initiation of treatment received a second course of Sb^V^ (20 mg/kg/day for 20 days). A third Sb^V^ course in combination with pentoxifylline (400 mg/kg/3 times daily) was administrated to patients who had persistence of the lesion 60 days after initiation of the second Sb^V^ course (Machado et al. 2002).

*LCL lesion and HS sample DNA isolation* - Bacterial DNA isolation was performed according to the PowerSoil DNA Isolation Kit (MO BIO) manufacturer’s instructions. DNA amounts were measured using the Qubit Quant-iT dsDNA BR Assay Kit protocol (Life Technologies, USA). Laminar flows and reagents used in DNA isolation were treated with ultraviolet before use to reduce the risk of contamination. DNA was submitted to V4 16S rRNA PCR amplification as described below.

*16S rRNA barcode library preparation and sequencing* - For each patient, two different amplicon libraries were prepared: one from LCL ulcers and the other from HS. The hypervariable V4 region of the 16S rRNA gene from each sample was amplified in triplicate using a primer set (515f/806r), as previously described (Caporaso et al. 2011, 2012). Amplification reactions were performed using the FastStart High Fidelity PCR System (Roche Applied Science, Germany) with a final volume of 25 μL containing 1.8 mM MgCl_2_, 200 μM dNTP, 0.4 μM primer (515f and 806r), 2.5 U FastStart High Fidelity Enzyme Blend, and a 100 ng/μL DNA sample. The PCR conditions were as follows: an initial step of 3 min at 94ºC, 35 cycles consisting of 45 s at 94ºC, 60 s at 50ºC, and 90 s at 72ºC, and a final step of 10 min at 72ºC. Amplicons were visualised using 1.5% agarose gels and ethidium bromide nucleic acid gel stain in a 0.5x tris-borate-ethylenediamine tetraacetic acid buffer. To confirm that the PCR reagents were not source of bacterial sequences, no-template control PCR was performed. No visible amplification signal was observed in this negative control on a gel, indicating that bacterial contamination was minimal.

The triplicate PCR products were pooled and purified using the PureLink PCR Purification Kit (Life Technologies), according to the manufacturer’s instructions. Following purification, each library was normalised to ensure equal library representation in pooled samples. The final libraries were pooled at equimolar concentration, denatured, and mixed with PhiX control to increase sequence diversity. Finally, the prepared library was loaded onto an Illumina MiSeq clamshell style cartridge for sequencing.

*Processing and analysis of 16S rRNA sequences* - After sequencing, all 16 rRNA raw sequence data were demultiplexed, quality-filtered, and analysed using Quantitative Insights Into Microbial Ecology (QIIME) v.1.7.0 pipeline (Kuczynski et al. 2012). After assignment of sequences, primer and tag sequences were removed before operational taxonomic unit (OTU) clustering. OTU clustering was performed with a 97% similarity threshold. Taxonomic identities were assigned using the RDP classifier tool (v.10.28) with 0.97 confidence threshold using Greengenes Database (from May 2013). Different numbers of reads per sample were obtained. Therefore, the sequence data were rarefied at 400,000 sequences per sample to account for this variation for Chao and observed species index calculations (i.e., alpha diversity), and principal coordinates analysis (PCoA) was performed using the phylogeny-based unweighted UniFrac distance metric (i.e., beta diversity).

*Statistical analyses* - Identification of OTUs that were significantly different in abundance in LCL lesions and contralateral HS was performed by paired *t* test with Bonferroni correction. To determine whether any groups of samples contained significantly different bacterial communities, analysis of similarities (ANOSIM) was conducted. Using the unweighted UniFrac distance matrix, distances were grouped as “within group” or “between group”. Significance levels were calculated by comparing the R statistic against the distribution generated from 10,000 permutations of the randomised dataset. The data were entered into a custom database (Excel, Microsoft Corp) and analysed using Prism 5. Quantitative data were reported as mean ± standard deviation or median (1st and 3rd quartile). Categorical data were reported as percentage and 95% confidence interval. Within patient phylum and genus level differences between lesions and HS were compared using multiple Wilcoxon rank tests for paired data. A p-value less than 0.05 were considered to be statistically significant.

*Availability of supporting data* - Raw sequences were deposited in the National Center for Biotechnology Information Short Read Archive (ncbi.nlm.nih.gov/sra) and are available with the following accessions: SAMN03198271, SAMN03198273, SAMN03198274, SAMN03198275, SAMN03198276, SAMN03198277, SAMN03198314, SAMN03198315, SAMN03198316, and SAMN03198317.

*Ethics* - The Gonçalo Moniz Research Centre/Oswaldo Cruz Foundation Ethical Committee approved the study (IRB 000026120; CEP 339/2010). The Declaration of Helsinki protocols were followed and all subjects provided written consent prior to participation in the study.

## RESULTS

*Cohort description* - To evaluate bacterial communities in LCL lesions, 10 subjects presenting ulcerated skin lesions with circular contours, infiltrative borders painless, and background with crude granules, epidemiologically and clinically consistent with LCL (Costa et al. 2009), were included in the study. One patient showed multiple lesions located mainly in the lower limbs, while the other nine patients presented single ulcers at different body sites shown in Table. These sites are the most frequent associated to LCL lesions in our experience (data not shown). Table also reported the demographic data, clinical characteristics, results of*Leishmania* diagnosis tests, and LCL lesion outcome for the patients included in the study.

**TABLE:** Demographic and clinical data, results of *Leishmania*diagnosis tests and LCL lesion outcome from patients in the study

Patients	Age/ sex	Occupation	Years living in endemic area	Wounds (n)	Wound location	Wound size (mm)	DTH with *Leishmania*antigen	*Leishmania brasiliensis* PCR	Parasite isolation in culture	Parasite identification in histopathology	Sb^V^ course numbers to healing
1	44/ F	Domestic worker	44	1	Hand	10 x 15	POS	POS	NEG	NEG	1
2	48/ M	Agricultural worker	48	1	Middle of leg	20 x 17	POS	POS	NEG	NEG	1
3	39/ F	Agricultural worker	39	1	Thigh	25 x 25	POS	NEG	NEG	POS	1
4	28/ M	Agricultural worker	28	1	Shin	15 x 12	POS	POS	NEG	NEG	1
5	28/ M	Agricultural worker	8	1	Shin	9 x 7	POS	POS	NEG	POS	2
6	20/ M	Agricultural worker	20	1	Middle of leg	15 x 13	POS	POS	POS	NEG	1
7	23/ M	Agricultural worker	23	1	Shin	20 x 20	POS	NEG	POS	NEG	1
8	43/ M	Agricultural worker	43	1	Thigh	15 x 12	POS	POS	NEG	NEG	1
9	31/ M	Agricultural worker	31	1	Shin	15 x 15	POS	POS	NEG	NEG	2
10	39/ M	Agricultural worker	39	3	Middle of leg	10 x 7	POS	NEG	NEG	NEG	3

DTH: delayed type hypersensitivity; F: female; M: male; NEG: negative; PCR: polymerase chain reaction; POS: positive.

All subjects presented positive results in the DTH test with*Leishmania* antigen (Table). Parasites were isolated in culture from patients 3 and 5. Parasites were also detected in histopathological exams of patients 6 and 7. All patients, except 3, 7, and 10, tested positive in the *L. braziliensis* PCR (Weirather et al. 2011). Patient 10 was the only one that the direct tests (culture, histopathology, or PCR) were negative. However, this patient presented an epidemiological history compatible with leishmaniasis (living for 39 years in the endemic area in an agricultural working), positive results in DTH test, and multiple lesions in lower limbs clinically very characteristic of LCL.

In relation to the lesions outcome after treatment, seven patients had complete regression of the lesion after the first Sb^V^ course (Table). Three patients (5, 9, and 10) showed treatment failure after the first Sb^V^ course and received a second course of Sb^V^. Only for patient 10 was necessary a third Sb^V^ course in combination with pentoxifylline. All patients showed complete lesion regression after treatment, consistent with leishmaniasis infection.

*Analysis of 16S rRNA sequences -* To evaluate whether bacterial communities in LCL lesions differed from those found on HS, samples from 10 different laboratorial and clinical confirmed LCL lesions and from the corresponding contralateral HS of the same patient were sequenced using a high throughput amplicon sequencing methodology. Demultiplexed 16 rRNA raw sequence data were quality-filtered and analysed using QIIME. After the quality filter checks, 13,759,042 high quality reads were obtained: 7,823,305 reads were from HS and 5,935,737 reads from LCL lesions. The mean sequence number per sample was 687,952.1 ± 101,984 (range from 408,767-927,648). The average length of sequences was 251 bp. Thirty-one phyla and 862 OTUs were identified in Greengenes Database. The lesion sample reads from patient 9 were excluded from statistical analysis because almost 90% of sequences from this patient were not classified into any phyla or OTU.

**Fig. 1:**
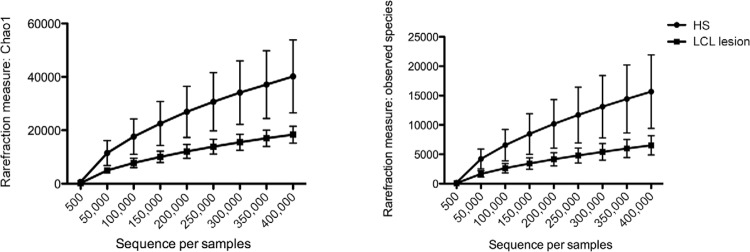
: rarefaction analysis for the Chao index (A) and observed species (B) of 16S rRNA sequences obtained from localised cutaneous leishmaniasis (LCL) lesions (square) and contralateral healthy skin (HS) (sphere). Lines represent the average of each group while the error bars represent the standard deviations.

*Alpha and beta diversity -* Rarefaction measurements (alpha diversity), shown in Fig. 1, demonstrated significant differences in observed species and Chao indices between the HS microbiome vs. LCL lesions denoting that HS presented significantly higher diversity levels compared to LCL wounds (p < 0.01).

PCoA plots based on unweighted UniFrac distance matrix (beta diversity), shown inFig. 2, exhibits separation between most HS and LCL lesion samples (ANOSIM, R = 0.58, p = 0.002) indicating that the LCL lesion microbiome differs from HS. PCoA also suggests that HS (red) has more samples with similar bacterial composition to each other than the LCL lesion. In addition, PCoA showed a mild cluster of samples of HS (red) related to the body sites where these samples were collected. Shin, thigh, and leg were grouped near to other samples from the same site.

**Fig. 2:**
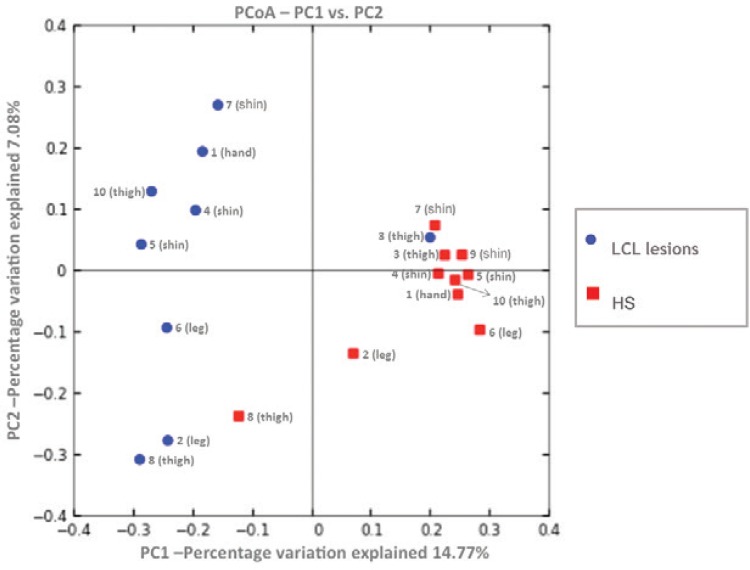
: principal coordinates analysis (PCoA) of unweighted UniFrac distances between localised cutaneous leishmaniasis (LCL) lesions (blue) and contralateral healthy skin (HS) (red) samples. The percentage of variation explained by each PCoA is indicated on the axis.

**Fig. 3:**
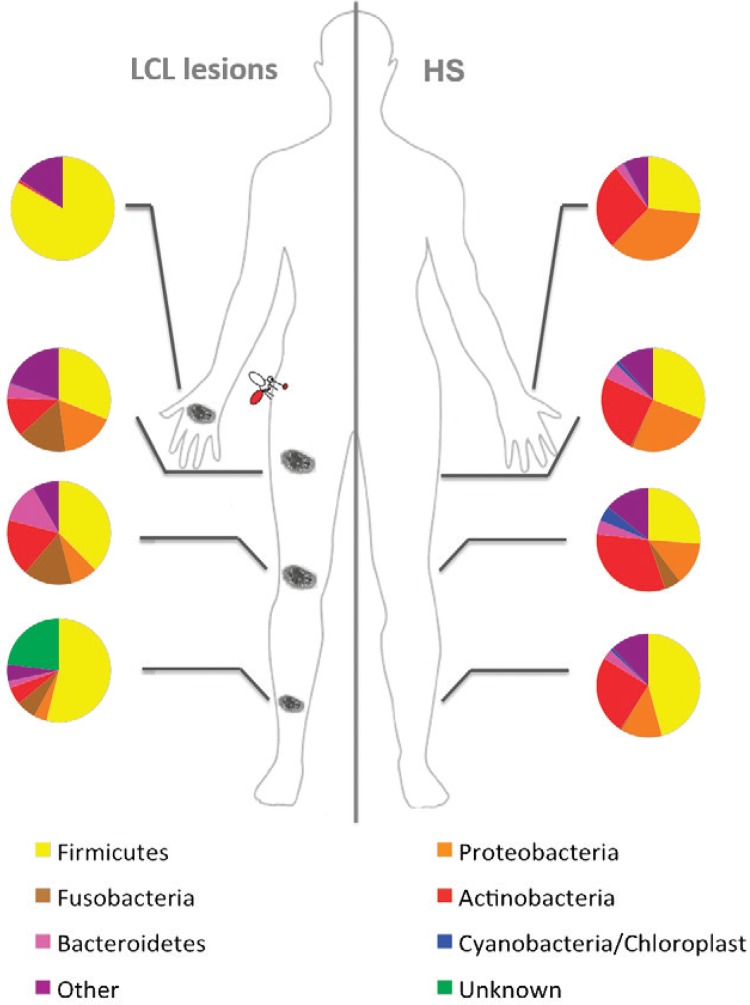
: topographical distribution of bacterial phyla detected in localised cutaneous leishmaniasis (LCL) lesions and healthy skin (HS) samples by high-throughput Illumina sequencing. Pie charts represent the major bacterial phyla found in different topographic body sites (hand, thigh, middle of leg, and shin).

*Differences in LCL lesions and HS microbial community at phylum level* - Thirty-one bacterial phyla were detected in both LCL lesions and HS. Most sequences detected in LCL lesions and HS were assigned to five phyla: Firmicutes, Actinobacteria, Proteobacteria, Fusobacteria, and Bacteroidetes. Chloroplast/Cyanobacteria were also detected in all HS samples and in LCL lesions from two patients. Fig. 3 shows the relative abundance of these phyla was variable considering the sampled skin sites from HS and LCL lesions. Unknown phyla in Fig. 3 represent sequences that have not been classified with the database used. This limitation occurs due the query insufficient alignment scores with the sequences database or low probability with RDP classifier.

**Fig. 4:**
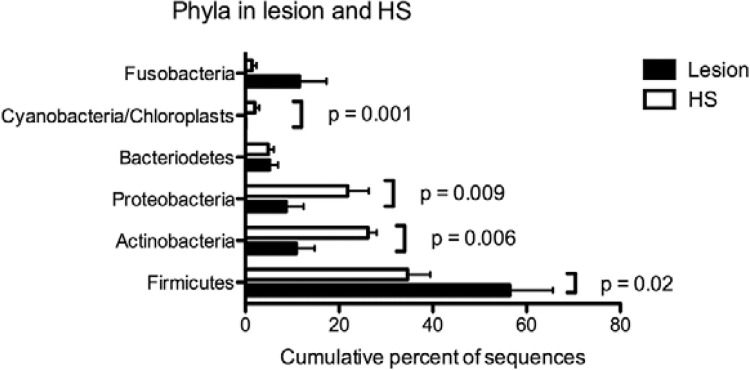
: cumulative percentages of sequences from bacterial phyla detected by high-throughput Illumina sequencing in healthy skin (HS) (white bars) and localised cutaneous leishmaniasis (LCL) lesion samples (dark bars) compared using multiple Wilcoxon rank tests for paired data.

Fig. 4 shows the cumulative percentages of sequences from bacterial phyla detected in HS and LCL lesions. The vast majority of sequences detected in LCL lesions were members of Firmicutes (54.3%), Actinobacteria (11.7%), Fusobacteria (11.6%), Proteobacteria (8.7%), and Bacteroidetes (5.1%). Firmicutes were more frequent in LCL lesions (54.3%) than HS (34.6%). This phylum’s abundance was significantly higher (p = 0.02) in LCL lesions. Actinobacteria and Proteobacteria were detected in LCL lesions, however with significantly lower percentages compared to HS (HS 26.2% vs. 11.7% LCL and HS 21.8% vs. 8.7% LCL, respectively). Cyanobacteria showed very low percentages in LCL lesions (0.1%) compared to HS (2%). The presence of this phylum in HS also differed significantly from LCL lesions (p = 0.001). One of the major phyla detected in LCL lesions was Fusobacteria, however without statistical significance. Bacteroidetes showed low percentages in both sites (LCL 5.1% vs. 4.9% HS).

**Fig. 5:**
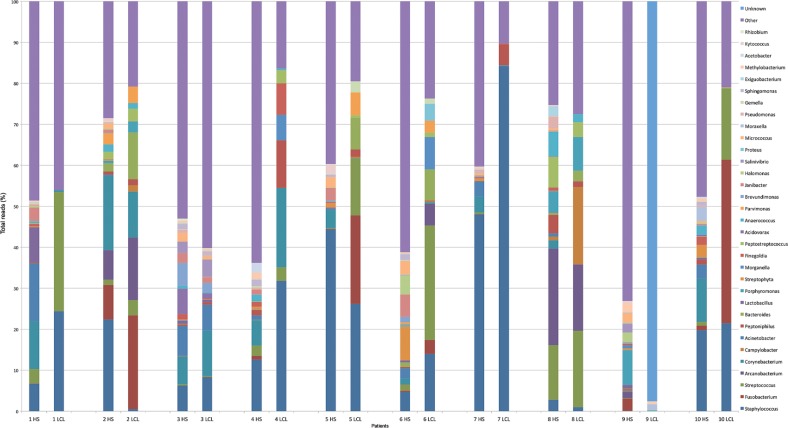
: relative abundance of most bacterial genera detected in 20 swab samples from healthy skin (HS) and localised cutaneous leishmaniasis (LCL) lesions by high-throughput Illumina sequencing. Bars represent the relative abundances between the majorities of genera detected in each sample.

*Differences in LCL lesions and HS microbiota at genus level* - About 862 bacterial genera were found in LCL lesions and HS, however the 33 genera demonstrated in Fig. 5 were the most common at both sites with relative abundances ranging the sampled skin sites (hand, middle leg, shin, and thigh) from HS and LCL. These were the bacterial genera which presented relative abundance greater than or equal to 2.5%.

**Fig. 6:**
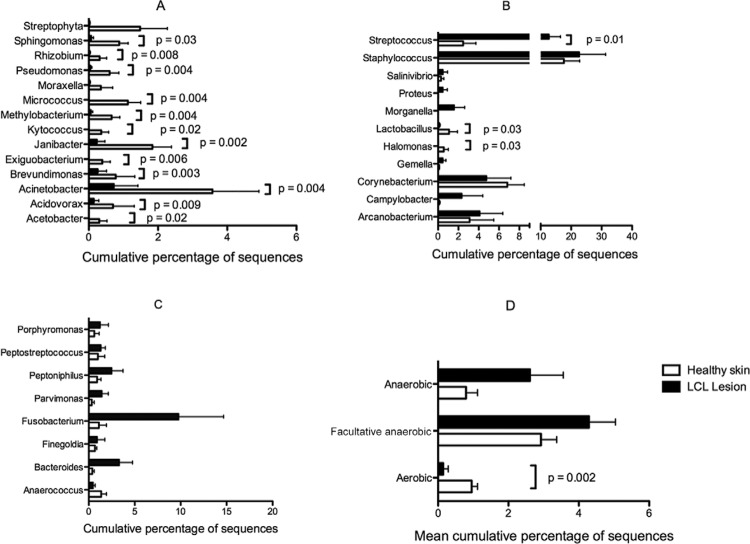
: bacterial genera detected by high-throughput Illumina sequencing in healthy skin (HS) (white bars) and localised cutaneous leishmaniasis (LCL) lesions (dark bars) samples compared using multiple Wilcoxon rank tests for paired data. A: cumulative percent of aerobic bacterial sequences in HS and LCL lesions; B: cumulative percent of anaerobic facultative and microaerophilic bacterial sequences in HS and LCL lesions; C: cumulative percent of strict anaerobic bacterial sequences in HS and LCL lesions; D: mean cumulative percent of sequences from anaerobic, facultative anaerobic/microaerophilic, and aerobic bacteria in HS and LCL.

Fig. 6 shows the cumulative percent of bacterial sequences in HS and LCL lesions and also the significant differences found in LCL lesions and HS microbiome. Bacterial genera were separated by the O2 tolerance to facilitate the preview. *Streptococcus* was the only genus found significantly increased in LCL lesions compared to HS (p = 0.01). Although not statistically significant, LCL lesions showed increased levels of strict and/or facultative anaerobic bacteria compared to HS. Moreover, about 15 environmental bacterial genera were significantly decreased in LCL lesions compared to HS, as shown in Fig. 6.

Considering the LCL lesions microbiome, the ubiquitous genera were*Staphylococcus* (present in all patients), followed by*Streptococcus* (detected in 8 of 9 patients),*Corynebacterium*, and *Peptoniphilus* (found in 7 of 9 patients), *Peptostreptococcus*, and*Fusobacterium* (observed in 6 and 5 patients, respectively).*Staphylococcus*, *Streptococcus*, and*Fusobacterium* were the predominant genera in lesion samples of patient 5 and 10, as observed in Fig. 5. These patients presented with a delayed healing process and took two and three cycles of leishmanicidal drugs, respectively, to complete the healing of the lesion.

## DISCUSSION

We compare the microbiological composition of laboratorial and LCL lesions with the contralateral HS microbiome from the same individuals using high throughput amplicon sequencing approach, which allows for deeper and broader insight into microbiological diversity. Our results showed that cutaneous microbiological signatures in human leishmaniasis lesions from patients living in an area with high predominance of *L. braziliensis* transmission (up to 95%) (Rosa et al. 1988) exhibit restricted bacterial diversity compared to those of HS. Three out of 10 patients did not show positive results in PCR. In these cases, it was not possible to identify the implicated*Leishmania*. Two of these patients did not show delayed healing, but presented parasites in histopathological examination (patient 3) and culture (patient 7). The negative results of patients 3 and 7 in the PCR could be explained by the presence of inhibitors in the isolated DNA of these patients, which hamper the amplification of target DNA in the PCR reaction. Different samples could also be used in the PCR and other tests (histology and culture) which caused this conflict in the results. Only patient 10 showed delayed healing and had negative results in the direct tests, suggesting that the PCR and other tests had insufficient sensitivity for this patient diagnosis. Although we did not identify other*Leishmania* species in these patients, it is likely they were infected with *L. braziliensis* or *L. amazonensis*. As only, these two species were identified in human cases from this endemic area. However, *L. amazonensis* is mainly associated with other clinical manifestation as DCL (Costa et al. 2009).

Both rarefaction measures and PCoA (alpha and beta diversity) results suggest that LCL and HS microbiome composition is different (Figs 1, 2). This result shows that compositionally intact HS bacterial colonisation is different from the opened wound with compromised epidermis caused by *Leishmania* infection. This microbiome composition variation in LCL lesions is probably because an open wound with compromised epidermis, associated with a subjacent inflammation caused by*Leishmania*, provides unbalance in indigenous microbial components (Scales & Huffnagle 2013). Our results suggest that the disturbance in LCL lesions is associated with an opportunistic contamination by commensal bacteria, which could resist to subjacent inflammation promoted by *Leishmania*, and develop against less adapted bacteria. In addition, this condition could provide pathogenic colonisation from different aetiologies (Scales & Huffnagle 2013).

Moreover, PCoA shows HS has more samples with similar composition to each other than the LCL lesion, probably because patients have similar microbiota related to the topographical body sites (Figs 2, 3). This site-dependent grouping based on the bacterial composition in the skin has already been described (Grice et al. 2009, Kong & Segre 2012).

The LCL microbiome showed lower diversity compared to HS. This restricted bacterial diversity in microbial community in affected compared to HS has also been reported in both inflammatory or not skin diseases, such as diabetic ulcers (Gontcharova et al. 2010), psoriasis (Alekseyenko et al. 2013), and atopic dermatitis (Kong et al. 2012). High bacterial diversity is associated with host protection, by innate and adaptive immune system modulation (Grice et al. 2010, Grice & Segre 2011, Kong & Segre 2012). The inflammation triggered by the innate immune system provides a suitable environment to some commensal bacteria, which could resist to the subjacent inflammation promoted by*Leishmania* and hampering development of less adapted bacteria.

Another explanation for the bacterial diversity reduction in LCL is the competition and cooperation behaviour in mixed microbial population, as bacteriocin and virulence factor production, which are commonly described in interspecies bacterial competition (Scales & Huffnagle 2013). Most adapted bacteria could influence development of another bacterium in a LCL environment.

We observed in HS samples a similar composition to the skin microbiome composed by the following phyla: Actinobacteria, Firmicutes, Bacteroidetes, and Proteobacteria (Grice & Segre 2011). Despite being less frequent, Cyanobacteria and Fusobacteria were also observed, suggesting microbiome composition variation for patients from our endemic area. Concerning the LCL microbiome, a similar microbial community was found in the microbiome of nonhealing diabetic foot ulcers, with similar percentages of Firmicutes (67%), Actinobacteria (14%), Proteobacteria (9.8%), Bacteroidetes (7.3%), and Fusobacteria (1.4%) at phylum level (Gardner et al. 2013).

Some aerobic bacteria presented decreased levels in LCL lesions. For instance,*Lactobacillus* and *Pseudomonas* play important protective roles on skin by the production of lactic acid, antimicrobial, and antifungal compounds, respectively (Cogen et al. 2008, Slover & Danziger 2008). However, many other bacteria detected in our study were not previously associated with the skin microbiome, that could be associated to high read number (687,952 reads mean per sample), allowing identification of new microbiome components.

We observed that anaerobes (*Fusobacterium*,*Bacteroides*, and *Peptoniphilus*), microaerophiles, and facultative anaerobic (*Streptococcus*,*Staphylococcus*, *Morganella*,*Campylobacter*, and *Arcanobacterium*) bacteria were most frequently detected in LCL (Fig. 6), with a reduction of the aerobic bacteria in normal skin. Similar results were observed in chronic wounds microbiome from several aetiologies, which presented a significantly larger proportion of anaerobes (*Bacteroidetes*,*Fusobacterium*), large quantities of Gram-negative rods such as*Pseudomonas*, *Proteus*, *E. coli*, and *Klebsiella*, and an increased proportion of*Staphylococcus* and *Streptococcus*. Chronic wounds also had a noticeably decreased proportion of protective coloniser in normal skin (Han et al. 2011).

These anaerobic bacteria may survive in the oxygen presence at LCL lesions, as observed in other wounds also exposed to air, by the symbiotically association with aerobic species. These aerobic bacteria could consume oxygen, creating localised low oxygen niches, allowing obligate anaerobes maintaining (Dowd et al. 2008, Gontcharova et al. 2010). Somehow, the infection-induced low oxygen microenvironment in LCL lesions could contribute with *Leishmania* persistence, once hypoxia negatively disturbs the killing of *Leishmania* due to type 2 NO synthase activity reduction of macrophages (Das & Lu 2014, Mahnke et al. 2014).

Analysing the differences in the skin microbiota at genus level, only*Streptococcus* was significantly higher in LCL lesion. This genus was previously linked to chronic ulcer infection (Issac-Márquez & Lezama-Dávila 2003, Shirazi et al. 2007,Dowd et al. 2008, Wolcott et al. 2009, Gontcharova et al. 2010) and healing delay (Davies et al. 2004, Bertesteanu et al. 2014). This suggests an important role of*Streptococcus* in chronic wounds maintenance, as LCL lesions.

Interestingly, we observed in two of three patients (patients 5 and 10) with delayed healing (take 2 or 3 Sb^V^ cycles) an association of three bacterial genera*Streptococcus*, *Staphylococcus*, and*Fusobacterium* in a higher frequency (Fig. 5). The other patient with delayed healing was excluded from analysis due to sequencing issues. These three genera were found in HS samples from the same patients; however, *Streptococcus* and*Fusobacterium* showed a lower prevalence in HS and presented a high increase in LCL lesions. These species alone seem not to influence healing time as observed in patients 1 and 2. These findings suggest a synergistic effect of these three genera on the healing process of LCL lesions. This was an interesting observation in our study; however it must be confirmed by further studies due the small number of analysed patients with delayed healing.

Ours results show the high bacterial community diversity in HS and LCL lesions. These findings demonstrate that traditional evaluation by culturing pathogenic bacteria is highly biased. The significant bacterial diversity reduction in LCL lesion microbiomes shows predominance of *Streptococcus* and strict or facultative anaerobes. It was also possible observe that microbial profile changes were directly related to the delayed healing in two out of the 10 patients. In these patients, *Streptococcus*, *Staphylococcus*, and*Fusobacterium* concomitant infection were implicated in a complex bacterial formation, leading to delayed healing. The present results provide insights about the influence of these specific bacteria on healing time, although this observation needs more investigation, due the sample size. Future investigations with larger samples are required to improve the microbiome understanding at different body sites. Moreover, temporal analyses are necessary to monitoring the microbiome changes during the ulcer-healing course.
